# The use of comet assay in plant toxicology: recent advances

**DOI:** 10.3389/fgene.2015.00216

**Published:** 2015-06-30

**Authors:** Conceição L. V. Santos, Bertrand Pourrut, José M. P. Ferreira de Oliveira

**Affiliations:** ^1^Department of Biology, Faculty of Sciences, University of PortoPorto, Portugal; ^2^Laboratoire Génie Civil et géo-Environnement - Groupe ISALille, France; ^3^Laboratory of Biotechnology and Cytometry, Centre for Environmental and Marine Studies, University of AveiroAveiro, Portugal

**Keywords:** plant comet assay, genotoxicity, metal, phytocompounds, radiation, pollutants, nanoparticles, DNA damages biomarkers

## Abstract

The systematic study of genotoxicity in plants induced by contaminants and other stress agents has been hindered to date by the lack of reliable and robust biomarkers. The comet assay is a versatile and sensitive method for the evaluation of DNA damages and DNA repair capacity at single-cell level. Due to its simplicity and sensitivity, and the small number of cells required to obtain robust results, the use of plant comet assay has drastically increased in the last decade. For years its use was restricted to a few model species, e.g., *Allium cepa, Nicotiana tabacum*, *Vicia faba, or Arabidopsis thaliana* but this number largely increased in the last years. Plant comet assay has been used to study the genotoxic impact of radiation, chemicals including pesticides, phytocompounds, heavy metals, nanoparticles or contaminated complex matrices. Here we will review the most recent data on the use of this technique as a standard approach for studying the genotoxic effects of different stress conditions on plants. Also, we will discuss the integration of information provided by the comet assay with other DNA-damage indicators, and with cellular responses including oxidative stress, cell division or cell death. Finally, we will focus on putative relations between transcripts related with DNA damage pathways, DNA replication and repair, oxidative stress and cell cycle progression that have been identified in plant cells with comet assays demonstrating DNA damage.

## Plant comet assay: general considerations

The first reports on the use of comet assay in plants date from the 1990's (e.g., Cerda et al., 1993; Koppen and Verschaeve, 1996; Navarrete et al., 1997; Koppen and Angelis, 1998).

Despite similarities with other eukaryotic systems, namely animal models, the comet assay protocols for plants take into account relevant differences including the presence of a rigid cell wall in plant cells. The localized presences of characteristic meristematic regions (e.g., the concentration of highly dividing cells in the root apex) and the fact that root is usually the organ directly in contact with contaminated soil and water, have also influenced the establishment of plant comet assays in ecotoxicological approaches. Technical details concerning plant comet assays in different organs and species have been thoroughly reviewed by Gichner et al. (2009).

For almost a decade, the comet assay remained restricted to some toxicological studies and to a few model species including *Allium cepa, Nicotiana tabacum*, *Vicia faba*, and *Arabidopsis thaliana* (for review, Gichner et al., 2009; Ventura et al., 2013).

Plant comet assay has been applied to an increasing variety of adverse conditions. Some recent reviews on this subject (Gichner et al., 2009; Ventura et al., 2013) revised most relevant advances in plant comet assay up to 5 years ago. Since then an increasing interest for comet assay in plants was shown (136 articles published between 2010 and March 2015 vs. 89 between 1995 and 2009). Therefore, here we will mostly emphasize most relevant advances within the last 5 years, and highlight current applications of this technique in plant (eco) toxicological studies. We will also discuss advances on genetic studies involving DNA damage and repair.

## Basic principles and methodologies

Comet assays traditionally use cell suspensions, which are embedded in agarose on a microscope slide, and exposed to lysis by exposure to detergent and high salt solutions (for review Collins et al., 2008; Azqueta et al., 2009). Lysis allows removing membranes and soluble cell components, leaving a supercoiled DNA nucleoid (Azqueta et al., 2011b). When submitted to electrophoretic conditions, DNA fragments will migrate toward the anode, forming a typical “comet tail.” The amount of strand breaks is overall proportional to the amount of DNA in the tail respectively to the DNA remaining in the head (Hovhannisyan, 2010).

However, in plants, the presence of a cell wall causes technical issues for performing the comet assay on plant tissues. To overcome these problems, a simple and efficient mechanical extraction to isolate cell nuclei was developed by Cerda et al. (1993), and then improved by Koppen and Angelis (1998), Navarrete et al. (1997), and Gichner and Plewa (1998). Since then, most of the researchers used directly those protocols or derived versions, such as described in Gichner and Plewa (1998). Recently, Pourrut et al. (2015) identified the key steps of comet assay in plants and proposed an optimized protocol to increase its reliability and its throughput. In the case of plant chopping, particular attention has to be paid to the presence of chloroplasts as they are important sources of free radicals and oxidative damage. For example, the first article on plant comet assay testing chemicals used isolated nuclei of *Vicia faba* root cells (Koppen and Verschaeve, 1996). In cellular assays, plants exposed to suspected genotoxicants are processed for nuclei isolation and analysis, whereas in acellular assays, nuclei from non-stressed plants are isolated and then incubated with the genotoxicants, before comet assay analysis.

The use of protocol variants allows detecting a wide range of DNA damages (see for review Angelis et al., 1999; Collins et al., 2008). Briefly, an alkaline treatment (referred hereafter as A/A) and electrophoresis at pH 13 or higher allows the detection of most single and double DNA strand breaks (SSBs and DSBs) and also alkali-labile sites. When the unwinding and subsequent electrophoresis are performed using a buffer pH~7–8, the comet assay is called “neutral” (N/N). A crucial difference is that at alkaline conditions, apurinic/apyrimidinic sites are more easily subjected to break (for details refer to Azqueta et al., 2011b). Other pH-variants (e.g., A/N) have meanwhile been introduced as alternative comet assays.

Moreover, the information provided by comets may also be increased by exposing the DNA to enzymes recognizing a specific lesion, e.g., formamidopyrimidine DNA glycosylase, Endonuclease III, thereby originating specific breaks. However, despite their strong interest and their early introduction in plant studies (Menke et al., 2000), these enzymes are still not much used in plants.

Comets may then be visualized by microscopy, by using a suitable DNA-binding dye, e.g., fluorescent dyes or silver staining. Data can be analyzed by visual scoring, ranging from 0 to 4 according to the damage class, or using computer-based image analysis (e.g., the software http://casplab.com/) that allows the quantification of several comet parameters, including the tail DNA %, tail length, tail extension moment or Olive tail movement (Azqueta et al., 2011b). Criteria for the best scoring approaches are however debatable (e.g., Azqueta et al., 2011a), but independently of the approach and scoring, it is consensual that this technique allows collecting data suitable for robust statistical analyses.

## Radiation

Plants are prone to DNA damage upon exposure to radiation from natural or anthropogenic sources. For this reason, the analysis of DNA damage in irradiated plants is a topic of growing interest and sensitive methods for detection of DNA damage have been applied (Table 1).

**Table 1:** **Overview of comet assay studies in plant toxicology**.

**Stress**	**Species**	**Tissue**	**Maximum dose**	**Nuclei**	**Comet type**	**Electrophoresis**	**Analysis**	**References**
**RADIATION**								
Light	*A. thaliana*	Leaves	1300 μmol m^−2^ s^−1^	Galbraith	A/A pH > 13	0.72 V/cm, 300 mA, 25 min, 4°C	(O)TM	Zeng et al., 2010
	*I. aquatica*	Roots	22 W m^−2^	PBS	A/A pH > 13	25 V, 300 mA 10 min, 4°C	IN_D_	Nishioka et al., 2010
	*R. sativus*	Cell suspension	430 W m^−2^	PBS	A/A pH > 13	25 V, 300 mA 10 min, 4°C	IN_D_	Ojima et al., 2009
UV	*A. thaliana*	Leaves	0.5 W m^−2^ UV-B, UV-A	Galbraith,T4endoV	A/A pH > 13	0.72 V/cm, 300 mA, 15 min, 4°C	(O)TM	Jiang et al., 2009
		Leaves	0.45 W m^−2^ UV-B, UV-A	Galbraith,T4endoV	A/A pH > 13	0.72 V/cm, 300 mA, 15 min, 4°C	(O)TM	Jiang et al., 2011
		Seedlings	3.5 kJm^−2^ UV-B	PBS-EDTA	N/N pH 8	1 V/cm, 12 mA, 5 min	%TD	Roy et al., 2011
	*N. plumbaginifolia*	Cell suspension	236 J mUV-C	PBS	A/A pH > 13	0.7 V/cm, 300 mA, 20 min	%TD	Abas et al., 2007
					N/N pH 8.4	2 V/cm, 10 mA, 2 min	%TD	Abas et al., 2007
	*S. polyrhiza*	Protoplasts	0.5 W m^−2^ UV-B, UV-A	Tris, T4 endoV	A/A pH > 13	0.72 V/cm, 300 mA, 15 min, 4°C	(O)TM	Jiang et al., 2007
γ-ray	(foodstuffs)	Various	5 kGy γ-ray	PBS Ca,Mg free	N/N pH 8.4	2 V/cm, 2 min	VS	Cerda et al., 1997
	(foodstuffs)	Various	10 kGy γ-ray	PBS-EDTA	N/N pH 8.4	0.66–0.83 V/cm, 300 mA, 5–40 min	%TD	Verbeek et al., 2008
	*(foodstuffs)*	Seeds	1 kGy γ-ray	PBS	N/N pH 8.4	2 V/cm, 2 min	TL	Koppen and Cerda, 1997
	*A. cepa*	Roots	4 Gy γ-ray	Sörensen(mod)	A/A pH > 13	0.65 V/cm, 230 mA, 20 min, 10°C	TL/HD	Navarrete et al., 1997
			50 Gy γ-ray	Tris	A/A pH > 13	0.65 V/cm, 230 mA, 20 min, 4°C	(O)TM	Saghirzadeh et al., 2008
	*A. thaliana*	Roots, leaves	3 Gy γ-ray	PBS	N/N pH 8.4	2 V/cm, 10 mA, 2 min, 4°C	VS	Vandenhove et al., 2010
		Seedlings	100 Gy γ-ray	PBS-EDTA	A/A pH 8.4	0.7 V/cm, 4°C	%TD	Moreno-Romero et al., 2012
	*H. vulgare*	Roots	110 Gy γ-ray	Sörensen(mod)	N/N pH 8	10 V/cm, 120 mA, 40 min, 4°C	%TD	Stoilov et al., 2013
					A/A pH 12.6	1 V/cm, 15 min, 4°C	%TD	Stoilov et al., 2013
	*M. truncatula*	Cell suspension	50 Gy γ-ray	Sörensen(mod)	N/N pH 8.4	1 V/cm, 8 min	VS	Donà et al., 2014
					A/A pH > 13	0.72 V/cm, 300 mA, 20 min, 4°C	VS	Donà et al., 2014
	*N. tabacum*	Roots, leaves	40 Gy γ-ray	Sörensen(mod)	A/A pH > 13	0.72 V/cm, 300 mA, 30 min, 4°C	(O)TM	Gichner et al., 2000
	*O. sativa*	Seedlings	200 Gy γ-ray	PBS-EDTA	A/A pH > 13	0.72 V/cm, 20 min, 4°C	VS	Macovei and Tuteja, 2013
		Seeds	200 Gy γ-ray	PBS-EDTA	N/N pH 8.4	1 V/cm, 8 min	VS	Macovei et al., 2014
	*P. x hybrida*	Roots, leaves	100 Gy γ-ray	Sörensen(mod)	A/A pH > 13	0.72 V/cm, 300 mA, 20 min, 4°C	(O)TM	Donà et al., 2013
	*P. nigra*	Cell suspension	300 Gy γ-ray	Sörensen(mod)	A/A pH > 13	0.75 V/cm, 300 mA, 30 min, 4°C	%TD,LDR	Nishiguchi et al., 2012
	*S. tuberosum*	Roots, leaves	30 Gy γ-ray	Tris	A/A pH > 13	0.74 V/cm, 300 mA, 15 min, 4°C	%TD	Gichner et al., 2008a
X-ray	*A. thaliana*	Leaves	15 Gy X-ray	PBS-EDTA	A/A pH > 13	300 mA, 15 min, 4°C	%TD	Enseit and Collins, 2015
	*N. tabacum*	Leaves, apex,	50 Gy X-ray	MBS-EDTA	A/N pH > 13/=8	1 V/cm, 10 mA, 5 min	%TD	Koppen et al., 1999
		Cotyledons			N/N pH 8	2 V/cm, 10 mA, 2 min	%TD	Koppen et al., 1999
					A/A pH > 13	1 V/cm, 300 mA, 5 min, 4°C	%TD	Koppen et al., 1999
	*O. sativa*	Calli	100 Gy X-ray	Sörensen(mod)	N/N pH 8.4	1 V/cm, 8 min	(O)TM	Endo et al., 2012
	*V. faba*	Roots	50 Gy X-ray	MES saline	A/N pH > 13/=8	1 V/cm, 10 mA, 10 min	%TD	Koppen and Angelis, 1998
					A/N pH 12.5/=8	1 V/cm, 10 mA, 10 min	%TD	Koppen and Angelis, 1998
					A/A pH > 13	1 V/cm, 10 mA, 10 min	%TD	Koppen and Angelis, 1998
		Leaves,apex,	50 Gy X-ray	MBS-EDTA	A/N pH > 13/=8	1 V/cm, 10 mA, 10 min	%TD	Koppen et al., 1999
		Cotyledons			N/N pH 8	2 V/cm, 10 mA, 2 min	%TD	Koppen et al., 1999
					A/A pH > 13	1 V/cm, 300 mA, 10 min, 4°C	%TD	Koppen et al., 1999
**METALS**								
Monovalent	*V. faba*	Roots, leaves	50 mg/L Tl(CH_3_COO)	Tris, C/A	A/A pH > 13	1 V/cm, 300 mA, 15 min, 4°C	(O)TM	Radić et al., 2009
Divalent	*A. cepa*	Roots	40 μM CdCl_2_	Tris	A/A pH > 13	0.72 V/cm, 300 mA, 25 min, 4°C	TL,(O)TM	Seth et al., 2008
			200 μM CdCl_2_	Tris	A/A pH > 13	300 mA, 20 min	%TD	Arya and Mukherjee, 2014
	*B. monnieri*	Roots, leaves	500 μM CdCl_2_	Tris, C/A	A/A pH > 13	0.72 V/cm, 300 mA, 30 min, 4°C	(O)TM	Vajpayee et al., 2006
	*L. sativa*	Roots, leaves	50 μM Cd(NO_3_)_2_	Tris	A/A pH > 13	0.74 V/cm, 300 mA, 30 min, 4°C	%TD,TL,(O)TM	Monteiro et al., 2012
	*L. luteus*	Roots	223 μM CdCl_2_	Tris-MgCl_2_	A/A pH 12.3	1 V/cm, 300 mA, 20 min, 8°C	TL	Arasimowicz-Jelonek et al., 2012
	*N. tabacum*	Roots, leaves	1.6 mM CdCl_2_	Tris, C/A	A/A pH > 13	0.72 V/cm, 300 mA, 30 min, 4°C	%TD,(O)TM	Gichner et al., 2004
			15 μM CdCl_2_	Tris	A/A pH > 13	0.8 V/cm, 300 mA, 20 min, 4°C	%TD	Tkalec et al., 2014
	*P. sativum*	Roots, leaves	7 (mg/kg) CdCl_2_	Tris	A/A pH > 13	0.72 V/cm, 300 mA, 30 min, 4°C	(O)TM	Hattab et al., 2010
	*S. tuberosum*	Roots, leaves	50 μM CdCl_2_	Tris	A/A pH > 13	0.74 V/cm, 300 mA, 15 min, 4°C	%TD	Gichner et al., 2008a
	*V. faba*	Roots	1 mM CdCl_2_	Honda	A/A pH > 13	1 V/cm, 300 mA, 10 min, 4°C	%TD,TL,(O)TM	Koppen and Verschaeve, 1996
			200 μM CdCl_2_	Tris	A/A pH > 13	300 mA, 15 min	%TD	Arya and Mukherjee, 2014
		Leaves	10 mg/L CdCl_2_·2.5 H_2_O	PBS-EDTA	A/A pH > 13	300 mA, 15 min, 4°C	VS	Lin et al., 2007
					A/N pH > 13/=8.4	4 min	VS	Lin et al., 2007
					N/N pH 8.4	15–17 mA, 6 min	VS	Lin et al., 2007
	*V. unguiculata*	Roots	10 mM CdCl_2_	Tris-MgCl_2_	A/A pH 12.3	1 V/cm, 300 mA, 20 min, 8°C	%TD,TL,(O)TM	Amirthalingam et al., 2013
	*N. tabacum*	Roots, leaves	50 μM ZnCl_2,_15 μM CdCl_2_	Tris	A/A pH > 13	0.8 V/cm, 300 mA, 20 min, 4°C	%TD	Tkalec et al., 2014
			80 mM Zn(CH_3_COO)_2_	Tris	A/A pH > 13	0.74 V/cm, 300 mA, 25 min, 4°C	(O)TM	Procházková et al., 2013
	*A. cepa*	Roots	3 ppm CuSO_4_, 11 ppm CoCl_2_	Tris-MgCl_2_	A/A pH 12.3	1 V/cm, 25 V, 20 min, 4°C	VS	Yıldız et al., 2009
			8 μM CuSO_4_	Galbraith	A/A pH > 13	0.72 V/cm, 300 mA, 15 min, 4°C	(O)TM	Qin et al., 2015
	*C. sativus*	Roots	11 ppm CuSO_4_	Tris-MgCl_2_	A/A pH > 13	24 V, 300 mA, 30 min, 4°C	(O)TM	İşeri et al.,^2011^
	*L. esculentum*	Roots	60 ppm CuSO_4_	Tris-MgCl_2_	A/A pH > 13	24 V, 300 mA, 30 min, 4°C	(O)TM	İşeri et al.,^2011^
	*M. truncatula*	Leaflets	0.2 mM CuCl2	Sörensen(mod)	N/N pH 8.4	1 V/cm, 8 min	VS	Faè et al., 2014
	*A. cepa*	Roots	100 μM Pb(NO_3_)_2_	Galbraith	A/A pH > 13	0.72 V/cm, 300 mA, 30 min, 4 °C	(O)TM	Jiang et al., 2014
			1 mM Pb(NO_3_)_2_	PBS	A/A pH > 13	25 V, 300 mA, 25 min	%HDNA, %TD, (O)TM	Kaur et al., 2014
	*N. tabacum*	Roots, leaves	2.4 mM Pb(NO_3_)_2_	Tris	A/A pH > 13	0.74 V/cm, 300 mA, 25 min, 4°C	(O)TM	Gichner et al., 2008c
	*T. triangulare*	Roots	1.25 mM Pb(NO_3_)_2_	Tris	A/A pH > 13	0.75 V/cm, 300 mA, 15 min, 4°C	(O)TM	Kumar et al., 2013
	*V. faba*	Roots	20 μM Pb(NO_3_)_2_	PBS-EDTA, C/A	A/A pH > 13	0.72 V/cm, 300 mA, 30 min, 4°C	%TD	Pourrut et al., 2011b
Trivalent	*A. thaliana*	Roots	3 mM B(OH)_3_	PBS-EDTA	N/N pH 8.4	1 V/cm, 15–17 mA, 6 min	%TD	Sakamoto et al., 2011
		Root	100 μM AlCl_3_	PBS-EDTA	A/A	0.6 V/cm, 250 mA, 25 min	(O)TM	Nezames et al., 2012
	*A. cepa*	Roots	200 μM AlCl_3_	PBS	A/A pH > 13	0.75 V/cm, 300 mA, 25 min, 4°C	TL	Achary et al., 2008
			800 μM AlCl_3_	Tris	A/A pH > 13	0.75 V/cm, 300 mA, 15 min, 4°C	(O)TM	Achary and Panda, 2010
				Tris, C/A	A/A pH > 13	0.75 V/cm, 300 mA, 15 min, 4°C	(O)TM	Achary et al., 2012a
				Tris	A/A pH > 13	0.75 V/cm, 300 mA, 15 min, 4°C	(O)TM	Achary et al., 2013
								Panda and Achary, 2014
	*H. vulgare*	Leaves	10 mM AlCl_3_	Tris	A/A pH > 13	0.75 V/cm, 300 mA, 15 min, 4°C	(O)TM;VS	Achary et al., 2012b
	*V. faba*	Roots	1 mM CrCl_3_	Honda	A/A pH > 13	1 V/cm, 300 mA, 10 min, 4°C	%TD, TL, (O)TM	Koppen and Verschaeve, 1996
Oxoanions	*A. cepa*	Roots	200 μM CrO_3_	Tris	A/A pH > 13	0.75 V/cm, 300 mA, 15 min, 4°C	(O)TM	Patnaik et al., 2013
	*P. sativum*	Roots, leaves	2 g/L K_2_Cr_2_O_7_	Tris	A/A pH > 13	0.74 V/cm, 15 min	%TD, (O)TM	Rodriguez et al., 2011
	*V. faba*	Roots, leaves	10 μM Na_2_HAsO_4_	PBS-EDTA	A/A pH > 13	300 mA, 15 min, 4°C	%TD, TL, (O)TM	Lin et al., 2008
		Roots	30 mg/L Na_2_HAsO_4_	Tris-NaCl	A/A pH > 13	25 V, 300 mA, 45 min	(O)TM	Boccia et al., 2013
			1 mM K_2_Cr_2_O_7_	Honda	A/A pH > 13	1 V/cm, 300 mA, 10 min, 4°C	%TD, TL, (O)TM	Koppen and Verschaeve, 1996
**NANOCOMPOUNDS**								
MWCNT	*A. cepa*	Roots	50 mg/L MWCNT	Tris	A/A pH > 13	26 V, 300 mA, 20 min, 4°C	%TD,VS	Ghosh et al., 2011
			10 μg/L MWCNT	Tris	A/A pH > 13	26 V, 300 mA, 20 min, 4°C	%TD	Ghosh et al., 2015a
Metal NPs	*A. cepa*	Roots, leaves	80 mg/L Ag NPs	PBS	A/A pH > 13	25 V, 30 min, 4°C	%TD	Ghosh et al., 2012a
	*B. rapa*	Roots, leaves	10 mg/L Ag NPs	PBS-EDTA	A/A pH > 13	35V, 300mA, 25min	%TD	Thiruvengadam et al., 2014
	*N. tabacum*	Roots, leaves	80 mg/L Ag NPs	PBS	A/A pH > 13	25 V, 30 min, 4°C	%TD	Ghosh et al., 2012a
Metal oxide NPs	*A. cepa*	Roots	100 ppm In_2_O_3_:SnO_2_ NPs	Tris-MgCl_2_	A/A pH > 12.3	1 V/cm, 25 V, 20 min, 4°C	VS	Ciğerci et al., 2015
			100 ppm Bi_2_O_3_ NPs	Tris-MgCl_2_	A/A pH 12.6	1 V/cm, 25 V, 20 min, 4°C	VS	Liman, 2013
			100 mg/L TiO_2_ NPs	Tris	A/A pH > 13	0.75 V/cm, 300 mA, 15 min, 4°C	(O)TM	Pakrashi et al., 2014
	*L. esculentum*	Roots	2 mg/ml NiO NPs	Galbraith, C/A	A/A pH > 13	0.7 V/cm, 300 mA, 30 min, 4°C	VS	Faisal et al., 2013
	*N. tabacum*	Roots, leaves	10 mM TiO_2_ NPs	Tris	A/A pH > 13	26 V, 300 mA, 20 min, 4°C	%TD,VS	Ghosh et al., 2010
Quantum dots	*M. sativa*	Cell suspension	100 nM CdSe/ZnS QDs	MES CaCl_2_/	A/N pH > 13/=8.4	25 V, 10 mA, 10 min, 4°C	VS	Santos et al., 2013
				FPG, EndoIII	N/N pH 8.4	25 V, 10 mA, 10 min	VS	Santos et al., 2013
**ORGANIC POLLUTANTS**								
Dyes	*A. cepa*	Roots	dyes of Petunia and	PBS, Tris	A/A pH > 13	0.7–0.75 V/cm, 300 mA, 20–25 min, 4°C	%TD,TL,%HDNA,(O)TM	Watharkar and Jadhav, 2014
			Gailardia	PBS, Tris	A/A pH > 13	0.7–0.75 V/cm, 300 mA, 20–25 min, 4°C	%TD,TL,%HDNA,(O)TM	Watharkar and Jadhav, 2014
Pesticides	*A. cepa*	Roots	100 ppm chlorfenvinphos 100 ppm fenbuconazole	Tris	A/A pH > 13	0.72 V/cm, 300 mA, 25 min, 4°C	VS	Türkoğlu, 2012
			100 ppm fenaminosulf	Tris-MgCl2	A/A pH > 13	1 V/cm, 20 min, 4°C	VS	Liman et al., 2013
			80 ppm imazethapyr	Tris-MgCl2	A/A pH > 13	1 V/cm, 20 min, 4°C	VS	Liman et al., 2015
	*I. balsamina*	Leaves	145 nM feranimol	Sörensen(mod)	A/A pH > 13	0.66 V/cm, 230 mA, 10 min, 4°C	LDR,VS	Poli et al., 2003
	*O. sativa*	Calli	5 mg/L aphidicolin	PBS-EDTA	N/N pH 8.4	6 min	(O)TM,VS	Kwon et al., 2013
	*P. vulgaris*	Roots	0.3 ppm 2,4-D, 0.3 ppm Dicamba	Tris-MgCl_2_	A/A pH 12.3	1 V/cm, 25 V, 20 min, 4°C	VS	Cenkci et al., 2010
Polyhalogenated	*A. cepa*	Roots	100 mg/L bromoform, 200 mg/L chloroform	Tris-MgCl_2_	A/A pH 12.3	1 V/cm, 25 V, 20 min	VS	Khallef et al., 2013
	*N. tabacum*	Roots, leaves	4.8 mM CBA, 1 mM DCBA, 0.48 mM TCBA	Tris	A/A pH > 13	0.72 V/cm, 300 mA, 25 min, 4°C	(O)TM	Gichner et al., 2008b
**CONTAMINATED MATRICES**								
Fly ash	*A. cepa*	Roots	fly ash mixtures (100%)	Tris	A/A pH > 13	0.7 V/cm, 300 mA, 20 min, 4°C	%TD,TL,(O)TM	Chakraborty et al., 2009
							%TD	Chakraborty and Mukherjee, 2011
			soil containing fly ash	Tris	A/A pH > 13	0.7 V/cm, 300 mA, 20 min, 4°C	%TD,(O)TM	Ghosh et al., 2012b
	*C. occidentalis*	Leaflets	soil containing fly ash	Sörensen(mod)	A/A pH > 13	0.72 V/cm, 300 mA, 30 min, 4°C	TL,VS	Love et al., 2009
	*V. zizanioides*	Roots	fly ash mixtures (100%)	Tris	A/A pH > 13	0.7 V/cm, 300 mA, 20 min, 4°C	%TD	Chakraborty and Mukherjee, 2011
Effluents	*A. cepa*	Roots	100% acid mine drainage	PBS-EDTA	A/A pH > 13	25 V, 300 mA, 20 min, 4°C	VS	Defaveri et al., 2009
								Netto et al., 2013
	*L. minor*	Plant	effluent waters	Tris	A/A pH > 13	1 V/cm, 300 mA, 20 min, 4°C	(O)TM	Radić et al., 2010
			fertilizer polluted water	Tris	A/A pH > 13	1 V/cm, 300 mA, 20 min, 4°C	%TD,(O)TM	Radić et al., 2013
Leachates	*A. cepa*	Roots	100% landfill leachate	Tris	A/A pH > 13	1 V/cm, 300 mA, 20 min	TL,(O)TM	Garaj-Vrhovac et al., 2013
	*E. fetida*	Shoots	100% landfill leachate	Tris	A/A pH > 13	25 V, 30 mA, 5 min, 4°C	(O)TM	Manier et al., 2012
	*T. repens*	Shoots	100% landfill leachate	Tris	A/A pH > 13	25 V, 30 mA, 5 min, 4°C	(O)TM	Manier et al., 2012
Metals	*N. tabacum*	Leaves	metal-polluted soil	Tris	A/A pH > 13	0.72 V/cm, 300 mA, 25 min, 4°C	(O)TM	Gichner et al., 2006
	*S. tuberosum*	Leaves	metal-polluted soil	Tris	A/A pH > 13	0.72 V/cm, 300 mA, 15 min, 4°C	(O)TM	Gichner et al., 2006
	*T. repens*	Leaves	metal-polluted soil	PBS	A/A pH > 13	300 mA, 15 min, 4°C	%TD, TL, (O)TM, VS	Bhat et al., 2011
Chemicals	*N. tabacum*	Leaves	PCB-polluted soil	Tris	A/A pH > 13	0.72 V/cm, 300 mA, 30 min, 4°C	(O)TM	Gichner et al., 2007
Radiation	*A. cepa*	Roots	19000 Bcq/kg ^226^Ra soil	Tris	A/A pH > 13	0.65 V/cm, 230 mA, 20 min, 4°C	(O)TM	Saghirzadeh et al., 2008
Gases	*P. tremuloides*	Leaves	1.5 × O_3_, 200 ppm above normal CO2	PBS-EDTA	A/A pH > 13	1 V/cm, 300 mA, 30 min, 4°C	%TD	Tai et al., 2010
**PHYTOCOMPOUNDS**								
	*A. cepa*	Roots	100 mg/l *T. turcica* extract	Tris-MgCl_2_	A/A pH > 13	1 V/cm, 20 min, 4°C	VS	Ciğerci et al., 2014
	*L. sativa*	Roots	200 μM epinodosin	Tris	A/A pH > 13	0.74 V/cm, 300 mA, 25 min, 4°C	%TD,TL,(O)TM	Ding et al., 2010a
			200 μM rabdosin B	Tris	A/A pH > 13	0.74 V/cm, 300 mA, 25 min, 4°C	%TD,(O)TM	Ding et al., 2010b
			5 μM narciclasine	Tris	A/A pH > 13	0.74 V/cm, 300 mA, 25 min, 4°C	%TD,(O)TM	Hu et al., 2014
	*P. alba*	Roots	100 nM saponins	PBS	A/A pH > 13	0.72 V/cm, 20 min, 4°C	VS	Paparella et al., 2015
					N/N	1 V/cm, 8 min, 4°C	VS	Paparella et al., 2015
	*R. sativus*	Radicles	100% extract J. regia	Tris	A/A pH > 13	25 V, 300 mA, 20 min, 4°C	%TD,TL,(O)TM	Petriccione and Ciniglia, 2012
**OTHERS**								
Osmostressors	*A. thaliana*	Seedlings	200 mM NaCl	PBS-EDTA	N/N pH 8	1 V/cm, 12 mA, 5 min	%TD	Roy et al., 2013
	*M. truncatula*	Roots	50 g/L PEG 6000	Sörensen(mod)	N/N pH 8.4	1 V/cm, 8 min	VS	Confalonieri et al., 2014
	*O. sativa*	Seedlings	100 mM NaCl	PBS-EDTA	A/A pH > 13	0.72 V/cm, 20 min, 4°C	VS	Macovei and Tuteja, 2013
**CONTROL MUTAGENS**								
	*A. cepa*	Roots, leaves	8 mM EMS	Tris, C/A	A/A pH > 13	0.72 V/cm, 300 mA, 20 min, 4°C	%TD	Bandyopadhyay and Mukherjee, 2011
	*A. thaliana*	Seedlings	50 mg/L BLM	PBS-EDTA	N/N pH 10	1 V/cm, 12 mA, 5 min	%TD	Böhmdorfer et al., 2011
			50 mg/L BLM	PBS-EDTA	N/N pH 8	1 V/cm, 12 mA, 5 min	%TD	Kozak et al., 2009
			8 mM MH	PBS-EDTA	A/A pH > 13	0.7 V/cm, 300 mA, 10 min, 4°C	%TD	Menke et al., 2001
			1 mg/L BLM, 5 mM MNU, 5 mM MMS, 8 mM MH, 0.5 mM MMC	PBS-EDTA	A/N pH > 13/=8.4	1 V/cm, 15–17 mA, 4 min	%TD	Menke et al., 2001
			1 mg/L BLM, 5 mM MNU, 5 mM MMS	PBS-EDTA	N/N pH 8.4	1 V/cm, 15–17 mA, 6 min	%TD	Menke et al., 2001
			2 μg/L BLM	PBS-EDTA	N/N pH 8.4	2 V/cm, 11 mA, 6 min	%TD	Wang et al., 2014
			2 μg/L BLM, 2 mM MMS	PBS-EDTA	A/N pH>13/=8.4	1 V/cm, 15–17 mA, 4 min	%TD	Waterworth et al., 2009
			2 μg/L BLM, 2 mM MMS	PBS-EDTA	N/N pH 8.4	0.6 V/cm,(20 V), 7 mA, 25 min	%TD	Waterworth et al., 2009
			50 μM BLM	PBS-EDTA	N/N pH 8.4	1 V/cm, room temperature	%TD	Moreno-Romero et al., 2012
	*B. monnieri*	Roots, leaves	100 μM MMS, 5 mM EMS	Tris, C/A	A/A pH > 13	0.72 V/cm, 300 mA, 30 min, 4°C	(O)TM	Vajpayee et al., 2006
	*C. capillaris*	Leaves	2 mM MH	Tris	A/A pH > 13	15 V/cm, 340 mA, 15 min, 4°C	TL,(O)TM,VS	Kwasniewska et al., 2012
	*H. vulgare*	Roots	200 mg/L BLM	Sörensen(mod)	N/N pH 8	10 V/cm, 120 mA, 40 min, 4°C	%TD,VS	Georgieva and Stoilov, 2008
			100 mg/L BLM	Sörensen(mod)	A/A pH 12.6	1 V/cm, 15 min, 4°C	%TD,VS	Georgieva and Stoilov, 2008
	*L. perenne*	Leaves	60 mM EMS	Tris-EDTA	A/A pH > 13	0.72 V/cm, 300 mA, 5 min, 4°C	%TD	Pourrut et al., 2015
	*M. giganteus*	Leaves	60 mM EMS	Tris-EDTA	A/A pH > 13	0.72 V/cm, 300 mA, 5 min, 4°C	%TD	Pourrut et al., 2015
	*N. tabacum*	Roots, leaves	8 mM EMS	Tris, C/A	A/A pH > 13	0.72 V/cm, 300 mA, 20 min, 4°C	%TD	Bandyopadhyay and Mukherjee, 2011
		Roots	10 mM EMS, 20 μM H_2_O_2_	Tris, C/A	A/A pH>13	0.72 V/cm, 300 mA, 30 min, 4°C	(O)TM	Gichner, 2003b
		Leaves	4 mM EMS, 0.4 mM ENU, 0.5 mM MH	Tris	A/A pH > 13	0.72 V/cm, 300 mA, 30 min, 4°C	%TD,(O)TM	Gichner, 2003a
			4 mM MH, 1 mM MNU	Tris	A/A pH > 13	16 V, 300 mA, 30 min, 4°C	%TD,(O)TM,VS	Juchimiuk et al., 2006
	*P. x hybrida*	Roots, leaves	3 mM EMS, 0.4 mM ENU	Sörensen(mod)	A/A pH > 13	0.72 V/cm, 300 mA, 20 min, 4°C	(O)TM	Donà et al., 2013
	*P. patens*	Protonema	50 mg/L BLM	PBS-EDTA	A/N pH > 13/=8.4	1 V/cm, 12 mA, 3 min	%TD	Holá et al., 2013
			50 mg/L BLM	PBS-EDTA	N/N pH 8.4	1 V/cm, 12 mA, 3 min	%TD	Holá et al., 2013
			50 mg/L BLM	PBS-EDTA	N/N pH 8.4	1 V/cm, 12 mA, 5 min	%TD	Kamisugi et al., 2012
	*S. tuberosum*	Roots, leaves	8 mM EMS	Tris	A/A pH > 13	0.74 V/cm, 300 mA, 15 min, 4°C	%TD	Gichner et al., 2008a
	*T. repens*	Leaves	60 mM EMS	Tris-EDTA	A/A pH > 13	0.72 V/cm, 300 mA, 5 min, 4°C	%TD	Pourrut et al., 2015
	*V. faba*	Roots	1 mM MMS, 1 mM EMS, 0.1 μM MMC, 1 mM CH	Honda	A/A pH > 13	1 V/cm, 300 mA, 10 min, 4°C	%TD,TL,(O)TM	Koppen and Verschaeve, 1996
			50 M BrdU, 1 M FdU	MBS-EDTA	A/A pH > 13	1 V/cm, 300 mA, 10 min, 4°C	%TD	Koppen and Verschaeve, 2001
		Roots, leaves	5 mM EMS	Tris-EDTA	A/A pH > 13	0.72 V/cm, 300 mA, 5 min, 4°C	%TD	Pourrut et al., 2015
	Various species	Leaves	10 mM EMS	Tris	A/A pH > 13	0.72 V/cm, 300 mA, 15–30 min, 4°C	(O)TM	Gichner et al., 2003

*C/A, cellular and acellular comets performed; A/A, alkaline variant; A/N, alkaline unwinding/neutral electrophoresis variant; N/N, neutral variant; IN_D_; DNA intactness; %TD, % tail DNA; TL, tail length; (O)TM, (Olive) tail moment; VS, visual scoring; %HDNA, % head DNA; LDR, length of tail-to-DNA ratio; A. cepa, Allium cepa; A. thaliana, Arabidopsis thaliana; B. monnieri, Bacopa monnieri; C. occidentalis, Cassia occidentalis; C. capillaris, Crepis capillaris; C. sativus, Cucumis sativus; E. fetida, Eisenia fetida; H. vulgare, Hordeum vulgare; I. balsamina, Impatiens balsamina; I. Aquatica, Ipomoea aquatic; L. sativa, Lactuca sativa; L. minor, Lemna minor; L. perenne, Lolium perenne; L. luteus, Lupinus luteus; L. esculentum, Lycopersicum esculentum; M. sativa, Medicago sativa; M. truncatula, Medicago truncatula; M. giganteus, Miscanthus giganteus; N. plumbaginifolia, Nicotiana plumbaginifolia; N. tabacum, Nicotiana tabacum; O. sativa, Oryza sativa; P. x hybrida, Petunia x hybrid; P. vulgaris, Phaseolus vulgaris; P. patens, Physcomitrella patens; P. sativum, Pisum sativum; P. alba, Populus alba; P. nigra, Populus nigra; P. tremuloides, Populus tremuloides; R. sativus, Raphanus sativus; S. tuberosum, Solanum tuberosum; S. polyrhiza, Spirodela polyrhiza; T. triangulare, Talinum triangulare; T. repens, Trifolium repens; V. zizanioides, Vetiver zizanioides; V. faba, Vicia faba; V. unguiculata, Vigna unguiculata*.

The effects of light excess on plant DNA using comet assay were firstly investigated by Ojima et al. (2009) on *Raphanus sativus* protoplasts. These authors demonstrated that light excess causes DNA degradations mediated by oxidative stress. In 2010, Nishioka et al. confirmed the role of reactive oxidative species (ROS) in light excess-induced DNA damages in *Ipomoea aquatica* root protoplasts, and correlated DNA damages observed by comet assay with chlorophyll degradation. However, these two studies did not take into consideration the potential role of UV in light-induced DNA damages. In a study designed to investigate UV-A and UV-B effects, Jiang et al. (2007) performed comet to detect specific DNA lesions as well as pyrimidine dimers formation (using T4 endonuclease V) in irradiated *Spirodela polyrhiza* protoplasts. These results were confirmed later in *Arabidopsis thaliana* root tip cells (Jiang et al., 2009, 2011). Jiang et al. (2011) also demonstrated that UV-B-induced DNA damage results in the delay of G1-to-S transition of plant cell cycle. However, by using a neutral comet assay (N/N variant), Roy et al. (2011) showed that UV-B-induced lesions were reversible, particularly in *A. thaliana* wild-type (Col-0), compared to DNA polymerase λ UV-B sensitive mutants. UV-C was also shown to induce both SSBs and DSBs in *Arabidopsis plumbaginifolia* protoplasts (Abas et al., 2007). These authors also highlighted the usefulness of the comet assay as an analytical tool for the analysis of repair kinetics in protoplasts. These results were confirmed by Bilichak et al. (2014) on *A. thaliana* protoplasts.

Besides natural exposure to radiation, plants are also irradiated for industrial purposes. For example, gamma (γ)-rays are used to increase seed vigor and/or enhance plant tolerance to environmental stresses. Navarrete et al. (1997) pioneered the comet research in plants through the optimization of different steps in the comet assay applied to γ-irradiated *A. cepa* roots. Moreover, Cerda et al. (1997), Koppen and Cerda (1997) and Verbeek et al. (2008) optimized the comet assay to screen DNA damage in γ-irradiated seeds, dried fruits and spices. At the same period, Gichner et al. (2000, 2008a) used the A/A variant to study the effects of the γ-rays in irradiated tobacco and potato plants, respectively.

Later, Böhmdorfer et al. (2011) used this technique to study DSB formation in *Arabidopsis* homologous recombination deficient mutants subjected to γ-rays. On the other hand, Vandenhove et al. (2010) applied low γ-radiation dose rates for long periods to *Arabidopsis* plants. Despite the growth limitations and induction of oxidative stress response, the low applied radiation dose applied did not induce DNA damages measurable by the comet assay. Moreover, Macovei et al. (2014) demonstrated the occurrence of DSBs in rice (*Oryza sativa* L.) seedlings after exposure to γ-rays concomitant with a difference in expression profiles of three miRNAs, and an increase of reactive oxygen species (ROS) levels. Combining the use of the comet assay, and the expression of genes encoding DNA repair-related proteins, Nishiguchi et al. (2012) investigated the mechanisms of γ-radiation-induced DNA degradation and repair in Lombardy poplar (*Populus nigra* var. *italica*). Donà et al. (2014) studied further the mechanisms associated with plant sensitivity to γ-irradiation. By comparison of A/N and N/N variants of the comet assay in *Medicago truncatula*, these authors argued that active repair of DSBs occurred in treated cells. However, SSB repair did not occur and SSBs continued to accumulate as a consequence of increasing ROS levels. It is necessary to point that the distinction by comet assay of DSBs and SSBs is not trivial, since the neutral assay with prolonged protease digestion at high temperature will more likely only detect DSBs. The research team demonstrated in *Petunia x hybrida* treated with low and high-dose γ-irradiation that the level of DNA strand breaks was higher in the high-dose group. However, after 2 h the two groups showed identical amounts of strand breaks, suggesting a faster initial DNA repair in the high-dose group.

Alkaline and neutral DNA comet assays were also used to estimate both the levels of DNA damages and the repair potential in the barley lines T-1586 and D-2946 after exposure to γ-rays and Li ions (Stoilov et al., 2013). The authors found that the mutant line D-2946 was more sensitive to γ-radiation, supporting that susceptibility to this radiation is genotype dependent. Overall, these data support that the genotype, radiation dose and time of radiation exposure are crucial factors that determine the effects of radiation on DNA integrity.

In comparison to γ-rays, comet assay has been little used to evaluate DNA damages induced by X-rays. Using alkaline comet assay, Koppen and Angelis (1998) demonstrated that X-rays induce a linear increase of DNA content in the comet tail of irradiated *V. faba* plants. Endo et al. (2012) reported that X-ray exposure in *calli* of *Oryza sativa* resulted in a dose-dependent increase of DSBs, as shown by neutral comet assay. Recently, Enseit and Collins (2015) studied the effect of low dose radiations on DNA repair mechanisms using alkaline comet assay. They identified two phases of DNA repair after acute exposures of 5 and 15 Gy (“rapid” and “slow” phases). With lower exposures (2 Gy and lower), they also highlighted that “rapid” repair was so fast that it was difficult to detect.

Concerning radioactive contaminations, Saghirzadeh et al. (2008) successfully demonstrated that very high levels of natural radioactivity (e.g., by accumulation of ^226^Ra) presented by soils were significantly genotoxic to *A. cepa* roots, with DNA damages measured by comet assay and compared to the effects of increasing γ-ray doses.

## Metals

Most of the contaminated sites worldwide are contaminated with heavy metals. In Europe, heavy metals contaminated almost 50% of the investigated sites (Panagos et al., 2013). Exposure to metals may induce a variety of direct and indirect phytotoxic effects (e.g., Silva et al., 2010). In general metals induce more severe symptoms in roots than in leaves, since roots are in direct contact with the soil and generally with the toxic contaminant.

The first comet assays evaluating metal genotoxicity in plants were pioneered by Koppen and Verschaeve (1996) which studied chromium (Cr) and cadmium (Cd) genotoxicity in *V. faba*. These authors showed a dose-dependent increase in DNA damage. More recently, Cd-induced DNA degradations were also observed in *Trifolium repens* (Bhat et al., 2011), *Lactuca sativa* (Monteiro et al., 2012), *Lupinus luteus* (Arasimowicz-Jelonek et al., 2012), *Vigna unguiculata* (Amirthalingam et al., 2013), *N. tabacum* (Tkalec et al., 2014), *V. faba* and *A. cepa* (Arya and Mukherjee, 2014). However, dose-dependent responses were not clearly observed in these studies. This could be explained by the fact that these authors lead hydroponic studies and used very high and environmental-unrealistic concentrations of cadmium. Monteiro et al. (2012) suggested that these high concentrations could induce Cd-DNA adducts that lead to DNA-DNA/DNA-protein cross-links, and/or formation of longer DNA fragments, and/or impairment of DNA repair mechanisms, which could explain these results. Interestingly, the only study using soil spiked with environmental-realistic concentrations of cadmium (Hattab et al., 2010), demonstrated a dose-dependent increase in DNA damages in *P. sativum*. Tkalec et al., (2014) and Amirthalingam et al. (2013) also used the comet assay to understand Cd-induced genotoxicity mechanisms. They suggested the implication of oxidative stress while Arasimowicz-Jelonek et al. (2012) showed that scavenging the endogenous nitric oxide (NO) pool during Cd stress, despite reducing the programmed cell death, did not affect the degree of DNA damages evidenced by comet assay. Recently, comet assay was used to investigate the difference of sensitivity to Cd exposure of *A. cepa* and *V. faba* (Arya and Mukherjee, 2014). The results indicated that exposure to Cd induced slight dose-dependent increase in chromosomal aberrations, DNA fragmentation and micronucleus frequency in both *A. cepa* and *V. faba*. However, *V. faba* appeared more sensitive than *A. cepa* toward Cd-induced genotoxicity, which was correlated to the increased level of oxidative stress in root tissues.

Along with Cd, aluminum (Al) genotoxicity has been the most studied during the last years. Achary et al. (2008, 2012a) and Achary and Panda (2010) demonstrated dose-dependent DNA damage induced by Al exposure on *A. cepa* roots. These results were confirmed later on *Hordeum vulgare* (Achary et al., 2012b) and *Andropogon virginicus* (Ezaki et al., 2013). These studies also highlighted the implication of oxidative stress in Al genotoxicity. Comet assay was also used to investigate the mechanisms of Al genotoxicity, underscoring the role of cell wall-bound NADH-PX in the Al oxidative burst-mediated (Achary et al., 2012a), and the role of signal transduction mediated by Ca^2+^ (Achary et al., 2013) and MAP Kinases (Panda and Achary, 2014) in Al-induced cell death and DNA damage. Interestingly, these authors also described the occurrence of adaptation responses that involved oxidative stress, and that root cells conditioned with low doses of Al (<10 μM Al^3+^) developed adaptive responses and protection mechanisms against genotoxic effects of the mutagenic agents methylmercuric chloride (MMCl) and ethyl methanesulfonate (EMS) (Achary et al., 2013). Moreover, the role of DNA damage in Al-dependent root growth inhibition was also investigated in *A. thaliana* mutants (Rounds and Larsen, 2008; Nezames et al., 2012).

The phytotoxicity of lead (Pb) including genotoxic aspects was reviewed by Pourrut et al. (2011a). Using comet assay, Gichner et al. (2008c) were the first to demonstrate dose-dependent Pb-induced DNA damage in *N. tabacum* in hydroponic and soil experiments. These results were confirmed on *Talinum triangulare* roots and correlated with Pb-induced oxidative stress (Kumar et al., 2013). However, both studies used very high and environmentally-unrealistic concentrations of Pb. More interestingly, dose-dependent Pb-induced DNA damage were also observed with lower and environmentally-realistic concentrations of Pb (<20 μM Pb) in *V. faba* plants (Pourrut et al., 2011b). Moreover, these authors also confirmed the role of oxidative stress in this damage process, since co-incubation with antioxidant vitamin E or the NADPH-oxidase inhibitor dephenylene iodonium inhibited DNA damage and micronuclei formation in exposed roots (Pourrut et al., 2011b). Recently, two studies performed on *A. cepa* confirmed the role of oxidative stress in lead-induced genotoxicity and that DNA damages are also tightly linked to the cell cycle (Jiang et al., 2014; Kaur et al., 2014).

Similarly, the micronutrient copper (Cu) was shown to induce significant DNA damages in *A. cepa* roots (Yıldız et al., 2009; Qin et al., 2015). Very high concentrations of copper chloride also increased DNA fragmentations in *P. sativum* roots but not in leaves (Hattab et al., 2010). Similarly to the above-cited metals, Cu-induced DNA damages were associated with cytotoxic damages involving oxidative stress in *Lycopersicon esculentum* and *Cucumis sativus* roots (İşeri et al., 2011) and other chromosome aberrations in *A. cepa* roots (Yıldız et al., 2009). Recently, Faè et al. (2014) used the neutral comet assay to demonstrate the overexpression efficiency of the DNA repair gene MtTdp2a for enhancing plant tolerance to Cu exposure in *Medicago truncatula* mutants.

By using the comet assay, Lin et al. (2008) proved that arsenate (10 μM) induced DNA damages in *V. faba* leaves and roots, in a dose-dependent manner and that these effects were associated with oxidative stress. Sturchio et al. (2011) confirmed As genotoxicity in *V. faba* roots grown on sandy and clay-loamy soil spiked with arsenate. In the same species, Boccia et al. (2013) combined the comet assay with infrared (FTIR), and near infrared (FTNIR) spectroscopy, to show that arsenate (20 and 30 mg/L) induced DNA damages which were associated with structural changes of different functional groups, suggesting the possible replacement of phosphate by arsenate in DNA.

The plant comet assay also contributed to clarify the effects of several other metals in plant DNA damages (Table 1). For example, Radić et al. (2009) demonstrated that the rare metal thallium (Tl), released to the environment as a by-product of Fe and Zn refining processes, induces DNA damages together with oxidative damages in *V. faba* seedlings. The comet assay was also helpful in demonstrating that boron (B) toxicity mechanism in plants involves DSBs and possibly replication blocks, with plant condensin II playing a critical role in DNA damages repair (Sakamoto et al., 2011). Rodriguez et al. (2011) and Rodriguez (2011) used a battery of genotoxic and cytotoxic biomarkers to assess Cr (VI) toxicity in pea, and were able to correlate Cr (VI)-induced DNA damages (demonstrated by comet assay) with cell cycle arrest at the G2/M checkpoint and with clastogenicity assessed by flow cytometry (Rodriguez, 2011, PhD thesis). Moreover, Patnaik et al. (2013) showed by alkaline comet assay that induction of DNA damage by Cr (VI) was dose-dependent in *A. cepa*. However, in plants exposed to 1-day treatment followed by 4-day recovery, no effects were found by comet assay. On the same plant species, cobalt (Co) was shown to induce significant DNA damages (Yıldız et al., 2009).

Besides some more established physiological analyses, the comet assay has also been conducted to determine the differential toxic effects affecting different plant organs. Procházková et al. (2013) showed that in *N. tabacum* zinc (Zn) induces higher DNA damages in roots compared to leaves. This differential effect was possibly attributable to the higher accumulation of Zn (II) in roots, compared to shoots. Tkalec et al. (2014) also observed these effects in *N. tabacum*. However, these authors also shown that, when Zn was added in the culture medium in combination with Cd, this metal conversely exhibited a protective effects against Cd-induced DNA damages.

It is worth noting that the interest of using the comet assay as a reliable biomarker on ecotoxicological assays is increasing, and Bandyopadhyay and Mukherjee (2011) applied both acellular and cellular comet tests to compare *A. cepa* and *N. tabacum* as toxicity models in rapid monitoring Cd-induced genotoxicity. Monteiro et al. (2012) used a battery of tests including the comet assay, to determine differences associated with organ dependence in Cd toxicity. The authors used *Lactuca sativa* and integrated cytostaticity/genotoxicity and oxidative stress data, where parameters measured by the comet assay (e.g., tail moment) were demonstrated to be relevant genotoxicity biomarkers. Despite still restricted to a few number, some studies have already used plant comet in field ecotoxicology assays of soils contaminated with metals (see Section “Contaminated Matrices” below).

## Nanocompounds

Plant comet assays are also increasingly used to assess the phytotoxicity of small-scale materials (Table 1), e.g., nanomaterials and in particular nanoparticles (NPs). Nanomaterials possess unique properties suitable for a wide range of industrial applications. For this reason and due to their intense uses and subsequent release to the environment, they are currently classified as emerging contaminants. One example of emerging nanomaterials are carbon nanotubes, that depending on the physical properties can pose cytotoxicity to mammalian and plant cells (Ghosh et al., 2011). Ghosh et al. (2011, 2015a) demonstrated a correlation between DNA strand breaks and the concentration of multi-walled carbon nanotubes in *A. cepa*, supporting the genotoxic potential of this type of nanomaterials.

The increasing amount of NPs in groundwater and soil has raised environmental concerns regarding their putative toxicity and fate through food chains. A large group of NP contaminants include toxic or reactive metals NPs. One of the most relevant pioneer studies of NPs genotoxicity in plants was done with TiO_2_ NPs in *A. cepa* (Ghosh et al., 2010). In this study the comet assay was used to assess DNA damages and this endpoint was combined with oxidative stress endpoints (e.g., malondialdehyde level). Moreover, in *A. cepa* roots, TiO_2_ NPs induced DNA damages confirmed by comet assay and correlated with the occurrence of chromosomal aberrations (Pakrashi et al., 2014).

Silver nanoparticles (AgNPs) were shown to induce DNA damages in *A. cepa* and *N. tabacum* with more pronounced effects in roots than in shoots (Ghosh et al., 2012a).

Recently, using higher NPs concentrations, Thiruvengadam et al. (2014) also demonstrated a dose-dependent increase in DNA damages in *Brassica rapa* ssp. rapa, and this result was confirmed by DNA laddering and TUNEL assays.

Bismuth (III) oxide NPs increased the nuclear DNA damages in *A. cepa* plants. These data supported the concomitant observation of chromosomal aberrations and mitotic aberrations in the same tissues (Liman, 2013).

The alkaline comet assay showed an increase of DNA damages in tomato seedlings exposed to NiO-NPs up to 2 mg/ml (Faisal et al., 2013). In this study the authors also used the plant comet assay test to assess the percentage of necrotic and apoptotic cells, however, these conclusions must be regarded carefully as the validity of the comet assay in identifying apoptotic cells remains a matter of discussion (Collins et al., 2008).

Indium (III) oxide and tin (IV) oxide is a mixture widely used in industrial coating. A significant increase in DNA damages was recently observed of *A. cepa* root meristematic cells exposed to doses up to 100 ppm of indium tin oxide suspension (Ciğerci et al., 2015).

Besides metal oxide NPs, quantum dots form another type of nanomaterials increasingly prevalent in the environment. Quantum dots are nanomaterials used in electronics which possess semiconducting properties, composed for example of arsenic (As), selenium (Se) and tellurium (Te) in various proportions. Despite their increasing prevalence in the environment, the toxicity of quantum dots in plants is largely unknown. In a pioneer study, Santos et al. (2013) used a battery of tests and gene expression related with DNA repair, and demonstrated that 10 nM 3-mercaptopropanoic coated-CdSe/ZnS quantum dots were cytotoxic and genotoxic to *Medicago sativa* cells. In this and other pioneer studies, the comet assay can play a pivotal role as a tool to assess environmental impacts of suspected emerging nanocontaminants.

## Organic pollutants

Several researchers have used the comet assay to monitor DNA damages induced in plants by numerous organic pollutants (Table 1). The most common organic chemical contaminants include reactive compounds, e.g., alkylating agents, azo dyes, cyclic aromatic hydrocarbons and chemicals incorporated in pesticides and herbicides.

The comet assay was recently used to better understand the role of homologous recombination and genome stability during DNA replication. Comet assay was used to study, in alfalfa, broad bean, lentil, miscanthus, onion, potato, tobacco, sugar beet and wheat, how different agents including ethyl methanesulfonate (EMS) and/or H_2_O_2_ induce DNA damages (Gichner et al., 2008a; Bandyopadhyay and Mukherjee, 2011; Pourrut et al., 2015). Due to their dose-dependent genotoxic effects, EMS and H_2_O_2_ became largely used as positive controls in plant comet assays, providing further robustness to the assay (Gichner et al., 2008a; Bandyopadhyay and Mukherjee, 2011; Pourrut et al., 2015). Similarly, the dose-dependent induction of DNA damages by compounds such as N-methyl-N-nitroso-urea (MNU), methyl methanesulfonate (MMS) and mitomycin C (MMC) (e.g., Menke et al., 2001; Juchimiuk et al., 2006) supported the wide use of these compounds as positive controls.

Azo dyes are important xenobiotic compounds, largely used in textile industry. Their putative genotoxicity was recently demonstrated in *Petunia grandiflora* and *Gaillardia grandiflora* by comet assay, in a pioneer study of plant–plant association for phytoremediation involving the treatment of textile dyes (Watharkar and Jadhav, 2014). Recently, it was demonstrated that bromoform (which may occur during disinfection processes of water) and chloroform (>25 μg/mL) increased chromosome aberrations and DNA damages, this last one assessed by comet assay in *A. cepa* roots (Khallef et al., 2013). Also chlorobenzoic acids (CBAs) may be found in soils contaminated with polychlorinated biphenyls (PCBs), and have mutagenic and carcinogenic effects in animals. Gichner et al. (2008b) demonstrated that the levels of CBAs inducing leaf withering or death also induced DNA migration in the comet assay.

In the last decade, several pesticides were demonstrated to induce DNA damages in plant cells (e.g., Poli et al., 2003). Endosulfan is an organochlorine pesticide widely used, and its genotoxicity was demonstrated in white clover (*Trifolium repens*) roots after exposure to doses up to 10 mg/L (Liu et al., 2009). The use of comet assay on *A. cepa* roots also demonstrated the genotoxic effects of the organophosphate insecticide/acaricide chlorfenvinphos and the triazole fungicide fenbuconazole (Türkoğlu, 2012). The experiment included tests/parameters such as the mitotic index, mitotic phase, chromosomal abnormalities, 2C DNA content (pg) and the plant comet assay on root meristem cells of *A. cepa*. Results indicated a robust negative correlation between both pesticides-induced DNA damage and 2C DNA amount. On the same plant model, Liman et al. (2013) studied the genotoxicity of the aromatic diazo fungicide and micro-biocide fenaminosulf. Comet assay clearly indicated a dose-dependent genotoxicity of Fenaminosulf in the root meristematic cells of *A. cepa*, which was confirmed by Mitotic index analysis. Herbicide genotoxicity was also evaluated by comet assay. Cenkci et al. (2010) demonstrated dose-dependent DNA-damages in common bean (*Phaseolus vulgaris*) roots used treated by two herbicides 2,4-D (2,4-dicholorophenoxyacetic acid) and Dicamba (3,6-dichloro-2-methoxybenzoic acid). These results were confirmed in the same study by RAPD analysis. Recently, Liman et al. (2015) also observed a dose-dependent DNA degradation induced by the imidazolinone herbicide Imazethapyr in *A. cepa* roots.

Antibiotics were also shown to induce DNA damages in plant cells. For example, the cytostatic effects of the antibiotic bleomycin (a DNA damaging glycopeptide) were demonstrated in plants, e.g., barley (Georgieva and Stoilov, 2008; Stoilov et al., 2013). Bleomycin also induced DNA oxidative damages and single and double strand breaks in the wild moss *Physcomitrella* lines and in the *lig4* mutant (Holá et al., 2013). Similarly, MMC induced a dose-dependent increase in DNA damages in *Arabidopsis* plants (Menke et al., 2001).

## Contaminated matrices

Despite the promising data concerning the robustness and suitability of the comet assay for screening metal-induced DNA damages in plant cells, its use to assess the genotoxicity of poly-contaminated matrices, including samples of contaminated soils, of leakages or fly ashes, remains scarce (Table 1). In a pioneer study, Gichner et al. (2006) used the alkaline comet assay to demonstrate DNA damages in both *N. tabacum* and *Solanum tuberosum* plants exposed to soils contaminated with a mixture of Cd, Cu, Pb, and Zn. Also, soil samples polluted with polychlorinated biphenyls were shown to induce DNA damages in tobacco plants (Gichner et al., 2007). These authors concluded that comet assays may be used for monitoring the DNA-damaging effects of environmental pollutants.

In a microcosm study, and using *T. repens* as plant model, Manier et al. (2012) found a dose-dependent increase in DNA damages in plants exposed to soil contaminated with landfill leachate. Garaj-Vrhovac et al. (2013) used the comet assay to validate two new methods of leachate treatment, which induced less DNA damages in *A. cepa* roots than the untreated landfill leachate. Comet assay was also used to evaluate the efficiency of new treatment technology to decrease acid mine drainage genotoxicity. Defaveri et al. (2009) and Netto et al. (2013) used *A. cepa* roots, and different biomarkers including DNA damages and other cytotoxic and physiological biomarkers, while Radić et al. (2010) used the aquatic species *Lemna minor*. In a previous study, these authors demonstrated in *Lemna minor* that the tail moment assessed by the plant comet assay and parameters related to oxidation were suitable as biomarkers for environmental monitoring of the toxicity of industrial effluents in Croatia (Radić et al., 2010). Importantly, the same group (Radić et al., 2013) found comparable responses in fish and *Lemna minor* regarding DNA damage and oxidative stress, after exposure to polluted surface water contaminated by a fertilizer factory effluent rich in fluorides, metals, and polycyclic aromatic hydrocarbons. The authors highlighted that their results imply that conventional chemical analysis should be extended to genotoxicity/toxicity biological assays to better predict potential health hazard.

Fly ashes are generated during combustion, and include fine particles, with different sizes, rising to the atmosphere. Their complex constitution raised questions on their genotoxicity to animals and plants. Love et al. (2009) demonstrated, based upon comet assay results, that higher levels of DNA damages were found in leaves of *Cassia occidentalis* exposed to fly ash, compared to non-exposed controls. The authors suggested that these DNA damages might be associated with foliar concentrations of As and Ni absorbed from the fly ash. Ghosh et al. (2012b) studied the genotoxicity in *A. cepa* of soil samples contaminated with metal-rich fly ashes from a thermal power plant in India and concluded that the observed DNA damages could be correlated to the presence of toxic metals. Also, Chakraborty et al. (2009) studied the genotoxic effects of fly ash comparing the comet assay and the Allium test in this model species. The authors supported the combination of these two techniques in monitoring assays. The same group used the comet to validate the relevance of *Vetiveria zizanioides* as a good candidate for remediation of fly ash dumpsites (Chakraborty and Mukherjee, 2011). They demonstrated this plant could grow in the presence of fly ash without any genotoxic effects in comparison to A. cepa which exhibited a very high DNA degradation (>80%). Later, this research group used comet assay on *A. cepa* to monitor the remediation efficiency of *V. zizanioides* on fly ash amended soils (Ghosh et al., 2015b). They showed that this plant was able to strongly mitigate the genotoxic potential of these soils. These results were also confirmed by a reduction in micronuclei formation, binucleate cells and chromosomal aberrations.

The effects of air contaminants on plant DNA-damages have also been studied in the last years. For example, *Populus tremuloides* clones exposed to air enriched with O_3_ alone, or CO_2_ + O_3_ showed increased DNA damages levels above background as measured by the comet assay, but these effects were genotype dependent (Tai et al., 2010).

## Phytocompounds

A wide number of phytocompounds (including alkaloids, phenolic compounds, glycosides, flavonoids, anthocyanins, etc) may have cytotoxic and genotoxic effects or have protective roles against stressing conditions in a wide number of species, including humans. The way phytocompounds influence oxidative stress balances, and regulate programmed cell death pathways and cell cycle chekpoints, support their wide therapeutic use (e.g., Ascenso et al., 2013; Ferreira de Oliveira et al., 2014). Recently, the interest of using comet assay to monitor genotoxic effects of some phytocompounds on other plant species has emerged (Table 1). For example, Petriccione and Ciniglia (2012) demonstrated the occurrence of a dose-dependent accumulation of DNA damages in *Raphanus sativus* (radish) radicles treated with *Juglans regia* husk water extracts. It should be noted that the authors stressed the need of performing accurate and appropriate statistical evaluations of comet results, an emerging topic of discussion. Ciğerci et al. (2014) also used alkaline comet assay to demonstrate the genotoxicity of *Thermopsis turcica* extracts on *A. cepa* roots. They showed dose-dependent DNA damages which were confirmed by RAPD profile analysis.

The alkaloid narciclasine (extracted from *N. tazetta*) was recently shown to inhibit plant growth of *Oryza sativa*, *A. thaliana*, *Brassica rapa* or *Lactuca sativa* (Hu et al., 2014). The comet assay, complemented with the terminal deoxynucleotidyl transferase dUTP nick end labeling (TUNEL) assay, showed a narciclasine dose-effect response in lettuce seedlings, and this triggered DNA damages may involve increased oxidative stress (Hu et al., 2014). Contrarily, anthocyanins protected DNA integrity (detected by comet assay) in *Arabidopsis* plants during prolonged exposure to high-light (1300 mmol/m^2^/s) (Zeng et al., 2010).

Epinodosin, and rabdosin B, diterpenoids isolated from *Isodon japonica*, exhibited a biphasic dose-dependent effect on *Lactuca sativa* root growth. The inhibitory effects of both compounds found at higher doses was paralleled with an increase of DNA damages and an inhibition of root cell mitotic activity or retardation of the cell cycle, respectively (Ding et al., 2010a,b). Other terpenes (saponins) extracted from *Medicago sativa* were shown to induce SSBs and DSBs in *Populus alba* cell cultures (Paparella et al., 2015). Very interestingly, these authors demonstrated that for all 11 tested saponins, neutral comet assay resulted in similar DSBs patterns, indicating a general response to saponin-induced genotoxic stress, not related to the specific structure of these molecules. Differently, the evaluation of DNA damages performed with alkaline comet assay provided distinct profiles depending on the tested saponin.

Comet assay was also used to evaluate the effect of the phytohormone salicylic acid. Interestingly, Yan et al. (2013) demonstrated that salicylic acid can generate DNA damages in the absence of a genotoxic agent in *A. thaliana*, supporting that activation of DNA damage responses is an intrinsic component of the plant defense responses.

## Comet assay and putative genetic associations

The comet assay has contributed to elucidate the DNA repair mechanisms involved in the response to external stress factors. A variety of methodologies can be used to investigate DNA repair mechanisms in plants (Azqueta et al., 2009), the most common being the study of plants exposed to DNA mutagens and comparison of plant strains deficient in specific DNA repair pathways. Ionizing radiation and a variety of genotoxins specifically induce DSBs and are frequently analyzed together with the action of radiomimetic compounds, such as bleomycin (e.g., Menke et al., 2001; Waterworth et al., 2009; Böhmdorfer et al., 2011; Wang et al., 2014), zeocin (Nishiguchi et al., 2012), or MMS (e.g., Menke et al., 2001; Vajpayee et al., 2006; Waterworth et al., 2009). Other mutagens frequently used to study DNA repair and strand breaks include agents that induce point mutations, e.g., N-ethyl-N-nitrosourea (ENU), MNU, or EMS (e.g., Menke et al., 2001; Donà et al., 2013), and the DNA crosslinking agent MMC (e.g., Koppen and Verschaeve, 1996; Menke et al., 2001).

In the past, plant strains deficient in DNA repair pathways have been analyzed by comet assay for their DNA repair capability under specific genotoxic stress. The first observation of biphasic DSB repair in plants with extremly rapid first phase was by Kozak et al. (2009). This approach, led to the identification of *A. thaliana AtRad18* (*SMC6B*) and *AtRad21.1* (*SYN2*) as important effectors in early repair of DSBs, after treatment with bleomycin (Kozak et al., 2009). Also important, through the use of comet functional assays, Moreno-Romero et al. (2012) showed that *Arabidopsis* mutant plants quickly repaired the DNA damage produced by bleomycin and γ-rays, and that they showed preferential use of non-conservative mechanisms. Moreover, in *Arabidopsis* knock-down strains of DNA ligase I, Waterworth et al. (2009) found by neutral comet assay that the *LIG1* knock-down strains were less efficient in the repair of DSBs compared to wild-type, suggesting that the *AtLIG1* gene is involved also in DSB repair pathway.

Several transcripts related with DNA damage pathways, DNA replication, and repair, oxidative stress and cell cycle progression have been identified in plant cells associated with alterations in comet assay profiles. Some of the most relevant studies in wildtype plants are summarized in Table 2. For example, Endo et al. (2006) demonstrated that *Arabidopsis fas* mutants showed increased levels of DNA DSBs. The authors proposed that the induction of DNA DSBs and enhanced transcription of genes involved in Homologous Recombination (HR) might occur during S phase and stimulate HR in fas mutants. Also, levels of formed DSBs were compared in rice wild type plants vs. an aphidicolin-sensitive phenotype. Without aphidicolin treatment, both WT and *osrecql4-2* mutants produced very low levels of DSBs, but these increased in the mutants after treatment (Kwon et al., 2013).

**Table 2:** **Genes differentially expressed in comet assay positive plants**.

**Gene**	**Gene function**	**Expr**.	**Stress**	**Species**	**References**
BRCA1	HR—DSB repair, ATM pathway (DSB-inducible)	Up	γ-ray BLM boric acid	*A. thaliana A. thaliana A. thaliana*	Böhmdorfer et al., 2011 Wang et al., 2014 Sakamoto et al., 2011
CAP-G2 (HEB1)	Tolerance to DSB induction	Up	boric acid	*A. thaliana*	Sakamoto et al., 2011
CAP-H2 (HEB2)	Tolerance to DSB induction	Up	boric acid	*A. thaliana*	Sakamoto et al., 2011
FPG	BER; removal of oxidized purines	Up	CdSe/ZnS quantum dots	*M. sativa*	Santos et al., 2013
GMI1	HR—DSB repair, ATM pathway (DSB-inducible)	Up	γ-ray, BLM, MMC	*A. thaliana*	Böhmdorfer et al., 2011
GR1	HR—DSB repair, ATM pathway (DSB-inducible)	Up	BLM boric acid	*A. thaliana A. thaliana*	Wang et al., 2014 Sakamoto et al., 2011
KU70	NHEJ—DSB repair	Up	γ-ray, zeocin	*P. nigra*	Nishiguchi et al., 2012
KU80	NHEJ—DSB repair	Up	salt stress (NaCl)	*A. thaliana*	Roy et al., 2013
LIG4	NHEJ—DSB repair	Up	γ-ray, zeocin salt stress (NaCl)	*P. nigra A. thaliana*	Nishiguchi et al., 2012 Roy et al., 2013
OGG1	BER; removal of 7,8-dihydro-8-oxoguanine	Down	γ-ray	*P. nigra*	Nishiguchi et al., 2012
PARP1	DSB repair (ATM pathway); SSB repair (ATR pathway)	Up	boric acid	*A. thaliana*	Sakamoto et al., 2011
PCNA	DNA replication and repair	Up	γ-ray	*P. nigra*	Nishiguchi et al., 2012
Polλ	NHEJ; NER in response to UV; DNA replication	Up	UV-B salt stress (NaCl)	*A. thaliana A. thaliana*	Roy et al., 2011 Roy et al., 2013
RAD51	HR—DSB repair, ATM pathway (DSB-inducible)	Up	γ-ray, zeocin boric acid	*A. thaliana P. nigra A. thaliana*	Böhmdorfer et al., 2011 Nishiguchi et al., 2012 Sakamoto et al., 2011
RAD51A2	HR	Up	X-ray	*O. sativa L*.	Endo et al., 2012
TDP1β	Repair of topoisomerase I-mediated damages	Up	CdSe/ZnS quantum dots	*M. sativa*	Santos et al., 2013
TOP1β	Remove DNA supercoils: transcription, DNA replication, recombination	Up	CdSe/ZnS quantum dots	*M. sativa*	Santos et al., 2013
XRCC4	NHEJ—DSB repair	Up	γ-ray salt stress (NaCl)	*P. nigra A. thaliana*	Nishiguchi et al., 2012 Roy et al., 2013
APX	Detoxification of peroxide	Up	CdSe/ZnS quantum dots γ-ray	*M. sativa Petunia x hybrida*	Santos et al., 2013 Donà et al., 2013
SOD	Detoxification of superoxide	Up	CdSe/ZnS quantum dots	*M. sativa*	Santos et al., 2013
MT2	Metal binding, ROS radical neutralization	Up	γ-ray	*Petunia x hybrida*	Donà et al., 2013
CDKA1	Cell cycle regulation	Up	boric acid	*A. thaliana*	Sakamoto et al., 2011
CYCA2;1	Cell cycle progression	Up	boric acid	*A. thaliana*	Sakamoto et al., 2011

*ATM, Ataxia telangiectasia mutated; ATR, ATM and Rad3 related; BER, base excision repair; HR, homologous recombination; NER, nucleotide excision repair; NHEJ, non-homologous end joining; DSB, double strand breaks; A. thaliana, Arabidopsis thaliana; M. sativa, Medicago sativa; O. sativa, Oryza sativa; P. nigra, Populus nigra*.

Böhmdorfer et al. (2011) studied the involvement of γ-irradiation and MMC induced one protein (GMI1), a structural-maintenance-of-chromosomes-hinge domain-containing protein in mechanisms of somatic homologous recombination in *Arabidopsis* mutant lines. Comet assay demonstrated that the *gmi1* mutants had a reduced rate of DNA DSB repair during the early recovery phase after exposure to bleomycin. Also Yao et al. (2013) used the comet assay to show an increase of DNA damage levels in *Arabidopsis sdg2* mutants, containing a mutation at SET DOMAIN GROUP 2, necessary for global genome wide deposition of histone H3 lysine 4 trimethylation in chromatin. With these results, authors contributed to elucidate the regulation of SDG2-mediated H3K4me3 on chromatin structure and genome integrity in plants.

Sakamoto et al. (2011) studied *Arabidopsis* mutants (*heb1-1* and *heb2-1*) hypersensitive to excess of boron (B). Excess of B induced DNA damages and affected the expression of *HEB1* and *HEB2*, which encode respectively the CAP-G2 and CAP-H2 subunits of the condensin II protein complex, important in maintenance of chromosome structure. These results suggested that DSBs are a cause of B toxicity and that condensin II reduces the incidence of DSBs (Sakamoto et al., 2011).

Santos et al. (2013) demonstrated in *Medicago sativa* that exposure to increasing concentrations of MPA-CdSe/ZnS quantum dots, led to an increase of DNA damages, and up-regulated the transcription of the DNA repair enzymes formamidopyrimidine DNA glycosylase, tyrosyl-DNA phosphodiesterase I and DNA topoisomerase I.

Roy et al. (2011, 2013) reported that *Arabidopsis atpolλ* mutant lines exposed to UV-B radiation or to high salinity and MMC treatment s showed higher accumulation of DSBs than wild-type plants and a delayed repair of DSBs. This fact suggested the requirement of Pol λ in DSB repair in plants. Gamma irradiated *Populus nigra* suspension-cultured cells showed increased levels of DNA damage and increase of the transcripts *PnRAD51*, *PnLIG4*, *PnKU70*, *PnXRCC4*, and *PnPCNA* while *PnOGG1* mRNA was repressed (Nishiguchi et al., 2012). On the other hand, Donà et al. (2013) tested genotoxic effects of γ-irradiation and found significant fluctuations on the levels of DSB and different capacities of DNA repair, together with dose-rate-dependent changes in the expression of the genes *PhMT2* (encoding for a type 2 metallothionein) and *PhAPX* (encoding for a cytosolic isoform of ascorbate peroxidase).

Probing FISH techniques have been successfully applied to comet assay preparations to detect specific DNA lesions, nuclear organizer regions (NORs) and telomeric regions in *V. faba* (Menke et al., 2000) or 5S/25S rDNA in *Crepis capillaris* (Kwasniewska et al., 2012).

Salt, drought and osmotic stress are ever more emerging as abiotic defies intimately related with soil overuse and climate changes (e.g., Santos et al., 2002; Brito et al., 2003). Salt stress induction of DNA damages has been explored in e.g., *Arabidopsis* mutants by Roy et al. (2013) who supported the role of Polλ in DNA damages repair. Salt stress and/or radiation induction of DNA damages was studied in rice by Macovei and collaborators who also evaluated the expression of *OsXPB2*, *OsXPD*, *OsTFIIS*, and *OsTFIIS*-like genes (Macovei and Tuteja, 2013; Macovei et al., 2014). Recently, Balestrazzi et al. (2014) demonstrated in *Medicago truncatula* plants that a prolonged exposure to osmotic stress can cause unwanted DNA damages, while negatively affected the expression profiles of genes involved in DNA repair, namely *MtTdp1* (tyrosyl-DNA phosphodiesterase), *top1* (DNA topoisomerase I), *MtTFIIS* (transcription elongation factor II-S) and *MtTFIIS*-like. So, despite comet assay has not been consistently applied to these environmental stresses in plants, the available data of their interference with DNA integrity, opens a perspective of their use in the near future. Also, Confalonieri et al. (2014) demonstrated that in *Medicago truncatula* the MtTdp2α-gene overexpression prevented the accumulation of DSBs in absence or presence of osmotic stress, and that the *MtMRE11*, *MtRAD50* and *MtNBS1* genes that are involved in DSB sensing/repair, being up-regulated in the *MtTdp2α*-overexpressing plants grown under physiological conditions, were no further up-regulated under osmotic stress (Confalonieri et al., 2014).

## Conclusions

In this review we have highlighted most relevant studies that used comet assay in plants to study the impact of stress conditions on plant DNA damages. This work was mostly focused on the most recent major advances in the last five, regarding conventional and emerging contaminants and complex matrices. The recent advances in the use of the plant comet assay to both a larger number of plant species, and a larger number of conditions, support the use of this technique as a robust and sensitive technique to assess DNA damages induced by stress conditions. Data also support that this simple and robust technique may be a powerful tool to complement conventional and -omics tools in situ environmental pollution monitoring. Moreover, new fields of research using plant comet assay are open, not only in environmental studies, but also in plant physiology, as this technique may help elucidating pathways involved in plant development, cell cycle/programmed cell death, or even plant disease resistance. Also, it remains an important field of research deciphering genetic mechanisms underlying processes related with DNA damage/repair, in which comet assay will have undoubtedly a crucial role.

### Conflict of interest statement

The authors declare that the research was conducted in the absence of any commercial or financial relationships that could be construed as a potential conflict of interest.
